# Factors Associated with Unfavourable Treatment Outcomes in Patients with Tuberculosis: A 16-Year Cohort Study (2005–2020), Republic of Karakalpakstan, Uzbekistan

**DOI:** 10.3390/ijerph182312827

**Published:** 2021-12-05

**Authors:** Jamshid Gadoev, Damin Asadov, Anthony D. Harries, Ajay M. V. Kumar, Martin Johan Boeree, Araksya Hovhannesyan, Lianne Kuppens, Askar Yedilbayev, Oleksandr Korotych, Atadjan Hamraev, Kallibek Kudaybergenov, Barno Abdusamatova, Bakhtinur Khudanov, Masoud Dara

**Affiliations:** 1 WHO Country Office in Uzbekistan, 16-Tarobiy, Tashkent 100100, Uzbekistan; kuppensl@who.int; 2 Center of Development of Professional Qualification of Medical Personnel, Tashkent 100007, Uzbekistan; damin_asadov@mail.ru (D.A.); atadjan@yandex.ru (A.H.); 3 Centre for Operational Research, International Union Against Tuberculosis and Lung Disease, 2 Rue Jean Lantier, 75001 Paris, France; adharries@theunion.org (A.D.H.); akumar@theunion.org (A.M.V.K.); 4 Department of Clinical Research, Faculty of Infectious and Tropical Diseases, London School of Hygiene and Tropical Medicine, Keppel Street, London WC1E 7HT, UK; 5 International Union Against Tuberculosis and Lung Disease, South-East Asia Office, C-6, Qutub Institutional Area, New Delhi 110016, India; 6 Yenepoya Medical College, Yenepoya (Deemed to be University), Mangaluru 575018, India; 7 Department of Lung Diseases, Radboudumc, 6500 HB Nijmegen/TB Expert Centre Dekkerswald, 6561KE Groesbeek, The Netherlands; Martin.Boeree@radboudumc.nl; 8 World Health Organization Regional Office for Europe, UN City, Marmorvej 51, DK-2100 Copenhagen, Denmark; hovhannesyana@who.int (A.H.); yedilbayeva@who.int (A.Y.); korotycho@who.int (O.K.); daram@who.int (M.D.); 9 Republican Phthisiology and Pulmonology Center Named after Sultanov, Nukus 1735401, Republic of Karakalpakstan; kallibek69@umail.uz; 10 Ministry of Health of Republic of Uzbekistan, Tashkent 100011, Uzbekistan; barno.abdusamatova@ssv.uz (B.A.); baxtinur@bk.ru (B.K.)

**Keywords:** tuberculosis, unfavourable treatment outcomes, central Asia, Republic of Uzbekistan, Republic of Karakalpakstan, drug resistant tuberculosis, death, failure, loss to follow up, transfer out

## Abstract

Tuberculosis (TB) remains a public health burden in the Republic of Karakalpakstan, Uzbekistan. This region-wide retrospective cohort study reports the treatment outcomes of patients registered in the TB electronic register and treated with first-line drugs in the TB Programme of the Republic of Karakalpakstan from 2005–2020 and factors associated with unfavourable outcomes. Among 35,122 registered patients, 24,394 (69%) patients were adults, 2339 (7%) were children, 18,032 (51%) were male and 19,774 (68%) lived in rural areas. Of these patients, 29,130 (83%) had pulmonary TB and 7497 (>22%) had been previously treated. There were 7440 (21%) patients who had unfavourable treatment outcomes. Factors associated with unfavourable treatment outcomes included: increasing age, living in certain parts of the republic, disability, pensioner status, unemployment, being HIV-positive, having pulmonary TB, and receiving category II treatment. Factors associated with death included: being adult and elderly, living in certain parts of the republic, having a disability, pensioner status, being HIV-positive, and receiving category II treatment. Factors associated with failure included: being adolescent, female, having pulmonary TB. Factors associated with loss to follow-up included: being male, disability, pensioner status, unemployment, receiving category II treatment. In summary, there are sub-groups of patients who need special attention in order to decrease unfavourable treatment outcomes.

## 1. Introduction

Tuberculosis (TB) is a communicable disease that is a major cause of ill health and one of the top 10 causes of death in lower-middle income countries. TB is caused by the bacillus Mycobacterium tuberculosis, which is spread when people who are sick with TB expel bacteria into the air; for example, by coughing. The disease typically affects the lungs (pulmonary TB) but can also affect other sites (extrapulmonary TB) [1].

TB remains a public health burden in central Asian countries, particularly in Uzbekistan. The World Health Organization (WHO) in 2019 estimated the TB incidence in Uzbekistan to be 69 per 100,000 population [1]. Uzbekistan is among the top 30 countries in the world with multidrug-resistant TB (MDR-TB, resistant to at least isoniazid and rifampicin) and one of the 18 high-priority TB burden countries in the WHO European Region [2].

The Republic of Karakalpakstan is a sovereign state within the Republic of Uzbekistan. The most serious concern is the continued spread of infectious diseases such as tuberculosis, hepatitis, and respiratory and diarrheal diseases in the Republic of Karakalpakstan [3]. The Republic of Karakalpakstan has seen the lowest TB treatment success rates when compared with other regions of Uzbekistan. A country wide cohort study of new and previously treated TB patients registered in the National TB Programme between 2005 and 2010 showed that the poor treatment success in the Republic of Karakalpakstan was due to high rates of death, patient loss to follow-up and treatment failure [4]. Drug resistance and its poor management is an important determinant of these adverse treatment outcomes. A drug resistance survey conducted in Uzbekistan in 2010 and 2011 demonstrated that 23% of new patients and 62% of previously treated TB patients had MDR-TB, respectively [5]. However, according to recent WHO estimates, the prevalence of MDR TB in the country has decreased and is now 12% in new patients and 22% in previously treated patients [1].

With decreasing levels of MDR-TB, it was important to assess whether these high rates of unfavourable treatment outcomes in the Republic of Karakalpakstan, which were demonstrated in 2005–2010, had declined. There was also a need to re-examine risk factors for unfavourable treatment outcomes. An analysis of region/district-wide data over a 16-year period therefore provided an important opportunity to conduct a region-level overview to identify the risk factors and potential interventions needed to improve treatment outcomes.

The aim of this study was to assess treatment outcomes and factors associated with unfavourable outcomes in TB patients registered in the republic between 2005 and 2020 and treated with first-line drugs. Specific objectives were to describe (i) the overall socio-demographic and clinical characteristics, (ii) assess their overall treatment outcomes and the annual trends in unfavourable treatment outcomes and (iii) determine the overall risk factors associated with unfavourable treatment outcomes over the 16-year period

## 2. Materials and Methods

### 2.1. Study Design

This was a cohort study using routinely collected data from registered TB patients who were initiated on treatment with first-line drugs between January 2005 and December 2020.

### 2.2. Setting

#### 2.2.1. General Setting

Uzbekistan is a double landlocked country situated in Central Asia with a population of over 34.5 million [6]. The country has 14 administrative divisions: 12 regions, one autonomous republic (Republic of Karakalpakstan, at the north-western end of the country) and one administrative city, the capital of Tashkent. The local administrative levels are districts and cities. Uzbekistan was part of the former Soviet Union and became independent in September 1991. Since then, it has embarked on several major health reforms covering health care provision, governance and financing, with the aim of improving efficiency while ensuring equitable access. Primary care in rural areas has been changed to a two-tiered system, while specialized polyclinics in urban areas are being transformed into general polyclinics covering all groups of the urban population [7]. The Republic of Karakalpakstan consists of 16 districts and one city [8], and in line with the rest of the country, has embarked on the same health reforms. Various factors such as water shortages, poor air quality and an unsustainable economy have together negatively affected the health status of the population and weakened the health care system [9].

#### 2.2.2. TB Control

In the Republic of Karakalpakstan, the TB programme is managed by the Ministry of Health of the Republic and the Republican TB and Pulmonology Center based in Nukus, the capital city of the republic. TB diagnosis and treatment are provided free of charge in all levels of care. These include: (a) the National TB Center, situated in Nukus, mainly receiving patients with complex disease, who need more specialized TB care and interventions; (b) district TB hospitals, where TB patients are able to receive inpatient TB treatment; (c) district TB dispensaries, mainly providing screening, laboratory diagnosis, outpatient treatment and treatment follow up services for TB patients, registered in their respective territories; and (d) primary health care facilities, where patients can seek health services, including screening for TB, as a first point of care. Private clinics do not provide any TB treatment [10].

After registration and initiation of treatment (category I and category III treatment for new cases and category II treatment for previously treated TB cases who have relapsed, failed or returned after loss-to-follow-up), patients are hospitalized during the intensive phase of treatment (two months for new cases and three months for previously treated TB cases); thereafter the continuation phase of treatment (four months for new patients and five months for previously treated cases) is provided in an outpatient mode. The composition and duration of the different treatment regimens are described in the footnotes of Table 1. Patients undergo sputum smear testing after treatment completion in the intensive phase and if the sputum smears are negative patients are referred to the district TB dispensary or primary health care facilities for the continuation phase of treatment. The continuation phase of treatment is usually managed as an out-patient by a TB dispensary or a primary health care facility, whichever is located close to the patient. Treatment is prescribed by TB specialists during the intensive phase and by primary health care workers during the continuation phase. The duration of both phases of treatment may be extended based on sputum smear results. During the continuation phase, patients undergo sputum smear examination at the 5th month of treatment and at the end of treatment.

People with presumptive TB are usually screened at the primary health care level and sputum specimens are sent to district TB laboratories for sputum smear microscopy. However, starting from 2016, Xpert MTB/RIF (a molecular diagnostic assay) has become the first test of choice in all regions of Uzbekistan [11]. In addition, it is now recommended that patients are considered for chest radiography and mycobacterial culture as part of the diagnostic work-up [12]. While people with TB are diagnosed and registered in the district TB dispensary, patients can choose between two types of treatment, the inpatient and outpatient modalities, respectively. Following the Soviet system of TB case management, all TB patients are encouraged to receive treatment in hospital, “the inpatient modality of treatment”. Eastern Europe and central Asian countries are incurring relatively high costs due to extensive use of hospitalization for patients in the intensive phase of treatment and a relatively long length of stay for people treated in hospital (an average of 58 days per person in 2019) [1]. Historically, first- and second-line drugs were procured by the project implementation unit of the Global Fund to fight AIDS, TB and Malaria. However, since 2016, Uzbekistan has started to allocate domestic funds for procuring first-line drugs and plans to further increase the government contribution for procuring second-line drugs. Drug-susceptible and drug-resistant TB patients receive anti-tuberculosis treatment in accordance with National TB treatment protocols which are aligned with the latest WHO recommendations. In some regions of Uzbekistan, short treatment regimens for drug-resistant TB are also being introduced as pilot projects, with this initiative having started in 2018 [12]. Standardized “conventional” treatment for MDR-TB has been available countrywide since 2013, being first started in the Republic of Karakalpakstan in 2003 with support from Medecins Sans Frontieres (MSF). MSF has supported MDR-TB treatment optimization by piloting a shorter nine to eleven month regimen since 2013 in the Republic of Karakalpakstan [13].

Treatment outcomes for TB patients are recorded mainly by district TB doctors: they indicate the outcome on special forms and report these to a higher level by filling the TB-08 form, for both drug-susceptible and drug-resistant TB patients. The terms and definitions of each treatment outcome are described in the national TB order #383, named “Strengthening of TB control activities in Republic of Uzbekistan”, which is aligned with the latest WHO recommendations on TB case management [14]. Definitions of treatment outcomes are provided in the footnotes of Table 2.

### 2.3. Study Population

All TB patients who were registered and who were initiated on first-line TB treatment in the Republic of Karakalpakstan between January 2005 and December 2020 were included in the study. During the study the districts of the Republic of Karakalpakstan were divided into three groups based on their geographical locations, which included: “Central part” (Chimbay, Nukus, Khujayli, Karauzak, Takhiatash, Bogataus districts and Nukus city), “North-West” (Kanlikul, Shumanay, Muynak, Kungrat and Kegeyli districts), and “South-East” (Amudarya, Takhtakupir, Beruniy, Ellikala and Turtkul districts) parts.

### 2.4. Source of Data, Data Collection and Data Variables

The source of data was the TB Electronic Surveillance and Case Management (TB ESCM) register, based on Epi Info 6, which contained individual patient data. Data variables were collected in relation to study objectives and exported into EpiData Analysis version 2.2.2.187 (EpiData, Odense, Denmark) and Stata version 12 (Stata Statistical Software: Release 12. StataCorp LP, College Station, TX, USA) for further analysis. Since 2005, all diagnosed patients are individually recorded in the TB register with a unique registration number. TB patients included in the study were given standardized first-line treatment and were monitored under routine programme conditions for treatment outcomes according to national and international recommendations. Unfavourable treatment outcomes included death, treatment failure, loss to follow-up and not evaluated (including transfer out); these outcomes were assessed together and separately. In univariable analysis, factors associated with unfavourable treatment outcomes were assessed using risk ratios (RR) with 95% confidence intervals and Poisson regression with robust standard errors. Variables with *p*-values < 0.1 in the univariable analysis were included into multivariable Poisson regression models, producing adjusted RRs. *p* values < 0.05 were regarded as significant. Variables showing a high proportion of missing values were excluded in the multivariable analysis.

## 3. Results

### 3.1. Characteristics of the Study Population and Their Overall Treatment Outcomes

Between 2005 and 2020, there were 36,121 TB patients registered in the Republic of Karakalpakstan. Of them, 797 (2%) had their treatment outcome missing (this included 783 patients registered in 2020 who were still receiving treatment), and 202 did not have a confirmed TB diagnosis; they were therefore excluded from the analysis. Table 1 shows the baseline demographic and clinical characteristics of 35,122 patients included in the study.

Two thirds of the patients were adults (*n* = 24,394, 69%), with smaller numbers of children (*n* = 2339, 7%) and adolescents (*n* = 2038, 6%). Fifty-one percent were male and nearly 70% lived in rural areas (*n* = 19,774, 68%). A small number of patients were HIV-positive (*n* = 19, <1%) or had a history of imprisonment (*n* = 9, <1%). A large majority had pulmonary tuberculosis (PTB) (*n* = 29,130, 83%), and more than twenty percent of patients had been previously treated (*n* = 7497, 21%). Nearly eighty percent had a successful treatment outcome (this includes patients whose treatment outcome is “cured” or “treatment completed”) at the end of treatment.

Table 2 shows the overall treatment outcomes of TB patients registered between 2005 and 2020. Almost 80% of patients had a favourable outcome (defined as treatment success). In the remainder, 12% either died, failed treatment or were loss to follow-up, while 8% transferred out.

### 3.2. Trends in Annual Treatment Outcomes between 2005 and 2020

**Figure 1:**
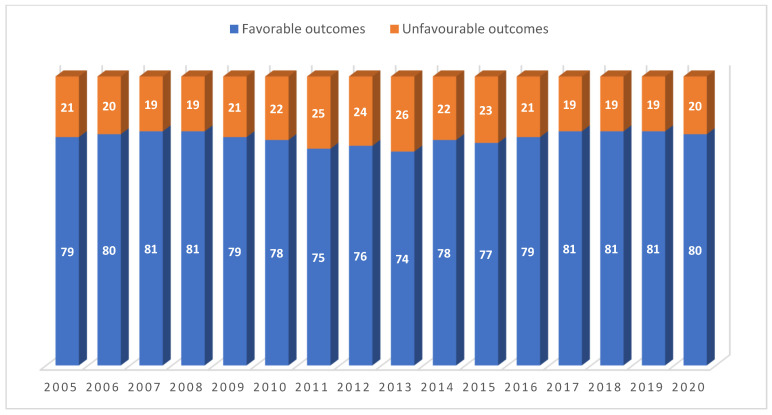
Proportion of TB patients with favourable and unfavourable outcomes recorded each year between 2005 and 2020 in the Republic of Karakalpakstan, Uzbekistan.

The annual trends in favourable and unfavourable treatment outcomes are shown in Figure 1. From 2006 to 2008, favourable treatment outcomes were at or just above 80%. There was a dip between 2011 and 2013, with favourable treatment outcomes ranging from 74% to 76%, and from 2017 onwards, favourable outcomes recovered to 80% or higher.

**Figure 2:**
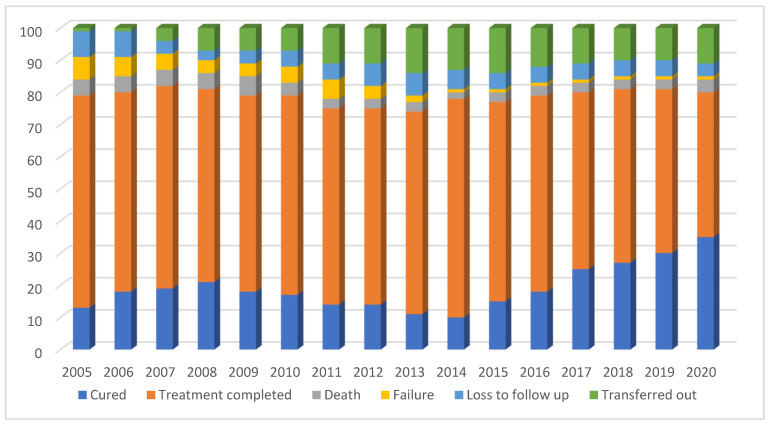
Proportion of TB patients with specific unfavourable outcomes, between 2005 and 2020 in the Republic of Karakalpakstan, Uzbekistan.

The annual trends in all six different treatment outcomes are shown in Figure 2. Cure rates were at their lowest between 2011 and 2013 and thereafter increased to above 30% by 2020. In the last five years compared with the first five years of the study period, death and failure decreased as a proportion of all outcomes. Loss to follow-up rates were approximately the same, while transfer out was higher in the last five years compared with the first five years.

**Figure 3:**
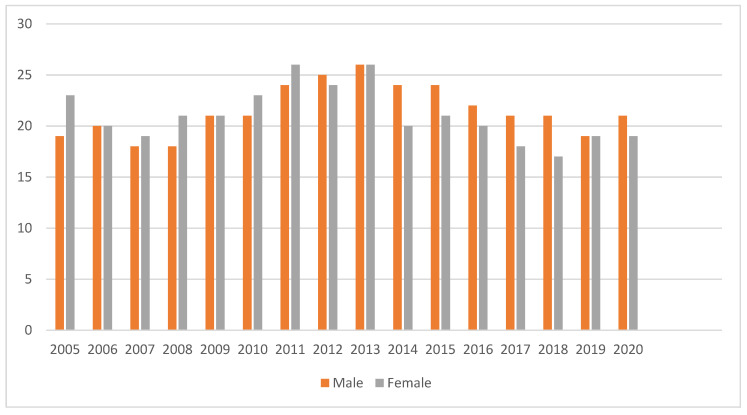
The annual trends of unfavourable outcomes disaggregated by sex, between 2005 and 2020 in the Republic of Karakalpakstan, Uzbekistan.

The annual trends of unfavourable outcomes disaggregated by sex are shown in Figure 3. Unfavorable outcomes were at their highest between 2011 and 2013 for both sexes. There was a significant decrease observed among females as compared to males up to 2018 followed by a slight increase by 2020.

### 3.3. Risk Factors for Unfavourable Outcomes, Death, Failure and Loss to Follow-Up

Risk factors for unfavourable outcomes are shown in Table 3. On adjusted analysis, unfavourable treatment outcomes were significantly higher in elderly patients (adjusted incidence rate ratio (aRR 2.3; 95% confidence interval (CI): 1.8–3.0), in patients residing in the North-West and Central parts of the republic (aRR 1.68; 95% CI: 1.5–1.8), in those with disability, pensioners, those who were unemployed, in patients who were HIV-positive, in patients who had pulmonary TB and in those who were initiated on category II treatment.

Risk factors for death are shown in Table 4. After adjustment of confounders, independent factors associated with higher death rates included: being elderly (aRR 5.2; 95% CI: 2.3–11); living in some parts of the republic; being a pensioner, being disabled (aRR 2.9; 95% CI: 1.92–4.48); being HIV-positive (aRR 9.5; 95% CI: 4.5–20); and receiving category II treatment (aRR 3; 95% CI: 2.6–4).

Risk factors for treatment failure are shown in Table 5. After adjustment of confounders, independent factors associated with treatment failure included: being adolescent (aRR 2; 95% CI: 1.2–3.4); being female (aRR 1.29; 95% CI: 1.1–1.5); having a history of contact with a TB patient (aRR 1.9; 95% CI: 1.6–2.4); and having pulmonary TB.

Risk factors for loss to follow up are shown in Table 6. After adjustment of confounders, independent factors associated with loss to follow-up were: being male, living in the central part of the republic (aRR 1.78; 95% CI: 1.5–2.1), being disabled, being a pensioner, being unemployed (aRR 1.95; 95% CI: 1.43–2.65), and receiving category II treatment (aRR 1.6; 95% CI: 1.3–1.9).

## 4. Discussion

This is the first comprehensive report on tuberculosis treatment outcomes over a sixteen-year period from the Republic of Karakalpakstan. Routinely collected data was used for describing the socio-demographic and clinical characteristics of registered TB patients, the overall treatment outcomes as well as the trends in treatment outcomes and, finally, factors associated with unfavourable treatment outcomes. These findings are important. While the WHO annually compiles aggregated data from countries and reports on TB data at the national level, detailed analyses of individual patient data as well as associations between unfavourable treatment outcomes and social-demographic and clinical characteristics are not reported [1].

Over the 16 years, the overall favourable treatment outcomes were good at almost 80%. The unfavourable outcomes of death, failure and loss to follow up were 5% or lower while transfer out was at 8%. In terms of trends in favourable outcomes, it was encouraging to see an increase in the proportion of patients cured, especially in the last five years. The reasons for this probably include (a) a countrywide scaling up of universal access to rapid molecular diagnostic tests, and (b) increasing treatment compliance with national treatment guidelines [11,14].

In terms of trends in unfavourable outcomes, the key findings were a decrease in the proportion of patients dying and being lost to follow-up, but an increase in those being transferred out. The decrease in deaths and loss to follow-up are probably because of better TB control efforts and treatment regimens over the years. The increase in transfer outs is of concern and points to a failure of communication between different ‘treatment units’ who transfer-out and receive transfer-in patients, but this needs to be studied further. With increasing use of mobile technology, this issue could be easily sorted out.

In the analysis of risk factors, adolescents [15,16,17,18] were considered in addition to children, adults, and the elderly, because they are a group that is particularly vulnerable with respect to infectious disease and yet are rarely assessed. [15]. Contrary to results observed in some other countries, in our study, adolescents had higher rates of treatment failure. Data from the Republic of South Africa demonstrate that, despite high rates of TB/HIV among adolescents, TB treatment success exceeds 90% and 86% among patient groups aged 10–14 years and 15–19 years, respectively, which contradicts our study findings [16]. Reasons for this are unclear and require further research. As reported from other studies, elderly patients had higher mortality rates [17]. This may be due to age-related factors, especially co-existing morbidities such as diabetes mellitus [18], immunosuppression and anaemia, as well as an increased tendency to having adverse drug reactions [18,19,20]. Chih-Hsin Lee et al. stated that in Taiwan the higher mortality rate among elderly patients was associated with a delay in seeking care at TB or other health facilities, resulting in many elderly patients presenting with advanced disease and thus a higher risk of death [21]. A study conducted in Germany found that co-morbidities were more common in elderly people compared to younger TB patients. TB treatment in elderly people follows established guidelines in the same way as for younger patients. However, the likelihood of drug-induced adverse effects and interactions with concomitant medications is increased [22].

Despite an almost similar gender distribution amongst the study population, the loss to follow-up rate in males was higher than in females. These data require further prospective studies to identify the potential reasons and the solutions. In contrast, treatment failure was higher in females compared with males. This is different to what was found in a previous study in Uzbekistan [4]. The reasons for this are again not clear and will require further research in prospective studies.

The range of unfavourable treatment outcomes, including death, loss to follow-up, and treatment failure varied in the different parts of the Republic of Karakalpakstan, with higher rates found mainly in the northwest and central parts of the republic. Such geographical differences have been described elsewhere, for example in Argentina, with respect to mortality [23]. High mortality rates were reported among patients from six northern provinces in Argentina. In those provinces, the researchers found poor adherence to TB treatment, high rates of HIV infection and AIDS, as well as high mortality among male and elderly patients. In Uzbekistan, the difference of treatment outcomes in districts is probably explained by a number of factors that include: (a) different rates of drug resistance; (b) the capacity of the district health systems to detect and treat cases early and follow them up, particularly in the continuation phase of treatment; (c) quality of primary health care services as a first point of screening of presumptive TB; (d) compliance with treatment guidelines (there was an increasing trend in using standardized regimens from 2% in 2012 to 44% in 2018 for the treatment of MDR TB patients [14]; and (e) patient characteristics such as migration and population mobility. Further qualitative research at the district level may clearly identify geographical factors associated with unfavourable treatment outcomes.

Our study also demonstrated that being unemployed, being a pensioner, and being disabled were strongly associated with unfavourable treatment outcomes. Unemployment is an important factor associated with unfavourable treatment outcomes and high rates of loss to follow-up were reported among patients without regular employment. Overall, 15,409 cases (44% of all TB patients in the study) were flagged as being unemployed in the Republic of Karakalpakstan over the 16 years. Researchers from Poland found that among the unemployed patients, radiological changes were often characterized by abnormal chest x-rays showing bilateral changes and numerous pulmonary cavities [24]. Other studies have confirmed these results, namely that unemployed patients present late and with advanced disease [25,26]. As a rule, patients may be unwilling to begin treatment because treatment and hospital stay affect household income. As a result, such patients delay presenting at health facilities, neglect the enormous beneficial role of TB treatment, and as a result experience long duration of treatment, the use of additional drugs and additional complications. Low levels of education and training, as well as unemployment, are all risk factors for poverty and social exclusion. Those unemployed are more likely to be lost to follow up and are therefore at increased risk of developing disease reactivation and anti-TB drug resistance. As a result, patients who are often income-earners with physically demanding side jobs become a financial burden on the household [27]. It has been recognized that housewife patients have a generally better nutritional status than unemployed patients, and poor nutrition increases the risk of developing active TB and having unfavourable treatment outcomes. This line of thinking is in line with studies conducted in Benin, Malawi, Nicaragua, and Senegal, which showed that women with TB were assessed and examined more frequently than men [27].

Our study also found that TB mortality among pensioners and the disabled was almost two to three times higher than in other groups of patients. With our analysis, it was not possible to attribute disability categories to nosological groups (conditions), and it was not possible to determine whether the disability was TB-induced or resulted from another condition. Our analysis showed that the ratio of the disabled to other groups varied by district. The lowest proportion of disabled patients was reported in Karauzyak district, while the highest proportion was reported in the Chimbay district. This means that with the average of 3% disabled in the Republic of Karakalpakstan, in Chimbay district the number of disabled patients enrolled in treatment was 70% higher than the average. Pensioners accounted for almost 14% of all notified patients in the register. Seventy-two percent of them completed treatment successfully, while the rest had unfavourable treatment outcomes. The most common unfavourable treatment outcome was death, which was almost twice as high as the average in the Republic of Karakalpakstan. This might be due to pensioners being elderly and having important co-morbidities such as diabetes mellitus.

HIV infection plays a special role in the development of unfavourable treatment outcomes. As estimated, people living with HIV with latent TB infection are about 20 times more likely to develop active TB compared with those who are HIV-negative [28]. Our study found that the TB death rate among HIV-positive patients in the period under review was almost 10 times higher than in HIV-negative patients. These data agree with studies conducted in other countries [29]. WHO recommendations for country TB and HIV programs to work together on timely initiation of antiretroviral therapy (ART) can considerably decrease unfavourable treatment outcomes, especially deaths among patients with TB/HIV co-infection [30,31].

This study also found that the mortality rate was relatively higher among patients with pulmonary TB, and the failure rate was about 10 times higher compared to patients with extrapulmonary TB. Data from Hong Kong showed that there was no difference in failure rates between pulmonary and extrapulmonary TB cases [32]. In the Republic of Karakalpakstan, the treatment success among extrapulmonary TB patients was 88%, while for pulmonary TB this rate was 77%. The reasons for this difference in Uzbekistan are not known and warrant further research.

Analysis of these groups showed that unfavourable treatment outcomes (death, loss to follow-up and treatment failure) were more frequent in patients who were initiated on category II treatment. Cases in which category II treatment was not effective (smear positive by the end of month nine of treatment)—were assigned to category IV treatment, i.e., to treatment of drug resistant TB, and they were registered in the MDR TB database. A systematic analysis, which included a review of 39 international articles published from 1999 to 2019, found that the proportion of patients that successfully completed category II treatment varied from 27% to 92%. In only two of the 39-studies was treatment success greater than 85%. Four of five studies that reported on HIV-infected patients demonstrated worse outcomes compared to HIV-negative retreatment cases. Only four studies reported that patients were found to be resistant to isoniazid, and treatment success rates varied from 11% to 78%. The review showed that successful treatment outcomes in category II patients were lower compared to patients in other treatment categories. The causes of the relatively low success rates were undetected drug resistance, co-morbidities, such as HIV, intolerance to anti-TB drugs, etc. [33]. High rates of unfavourable treatment outcomes in patients that were initiated on category II treatment in Uzbekistan could be associated with high levels of drug-resistant TB (MDR accounted for 23% of new cases and 62% of retreatment cases) in the country [5].

High rates of drug-resistant TB may be associated with the spread of the Beijing strain in Central Asia and the former Soviet Bloc countries. Shitikov E. et al. believe that the Beijing strain of Mycobacterium tuberculosis is associated with a higher risk of unfavourable treatment, including treatment failure and relapse in many Asian countries. The rapid global spread of the Beijing genotype is receiving increased attention because it can cause a higher risk of treatment failures [34]. The virulent strain in Central Asia is a branch of the Mycobacterium tuberculosis Beijing genotype that is associated with multidrug resistance, increased transmissibility and epidemic spread in some parts of the former Soviet Union. In addition, migration flows bring these strains far beyond their areas of origin [35]. Recent international experience shows that for MDR-TB patients without intolerance or resistance to main second-line drugs (i.e., fluoroquinolones), treatment can be considerably shorter, which can decrease the burden on patients and national TB programs in general. In recent years, the interest in reducing the duration of MDR-TB treatment has led to a number of initiatives to treat patients with shorter regimens under programmatic as well as trial conditions. When used in carefully selected MDR-TB patients who have not been previously exposed to nor have resistance to second-line drugs, these regimens have been reported to achieve relapse-free cures in over 85% of cases, even under programmatic conditions. In 2016, on the basis of data from observational studies of the shorter regimens in different Asian and African countries, the WHO recommended a standardized shorter MDR-TB regimen based on the regimens under study for eligible patients [12,36,37].

The main strength of this study relates to the large size and representativeness of the data. It is the first study ever conducted in the Republic of Karakalpakstan to assess risk factors associated with unfavourable treatment outcomes in patients starting on first-line drugs, and has used individual patient data rather than aggregate data as most national TB programs report.

However, there are several study limitations. First, the study was reliant on routinely collected data, which may have been subject to some reporting errors. Second, it was not possible to analyze some particularly interesting subgroups of patients, such as prison inmates, as large amounts of these data were incomplete. Third, unfavourable outcomes were not analyzed in relation to sputum smear results, as these data were not reliably recorded in the electronic database. Mycobacterial culture data were also not available in the electronic database. Fourth, the study patients were not categorized into “new patients” and “previously treated patients”, as is normally done. However, those treated with Category I regimens were mainly the new patients and those treated with Category II regimens were patients who had relapsed, failed treated or had returned to treatment after being lost to follow-up. Treatment outcomes in relation to these different treatment categories were evaluated. Finally, a major limitation was the misclassification of ‘transfer-outs’. Between 2003 and 2005, two MDR-TB pilot programs were started in Karakalpakstan and Tashkent city. Patients diagnosed with MDR-TB were transferred to the pilot clinics, and their records were transferred to the MDR-TB register. In many circumstances, these patients were classified in the national database as ‘transferred out’ rather than as ‘failure’. Furthermore, for patients who were transferred out to a different province, the national database should have been updated to reflect the final outcome for that patient, but this never happened. Therefore, in this study the outcome “transferred out” consisted of patients for whom the final outcome was not ascertained and patients who were transferred into the MDR-TB register after failing standard treatment between 2003–2005. As such, a proportion of the transfer outs were essentially treatment failures, although the size of this proportion remains unknown. Scale-up of short treatment regimens from pilot projects to programmatic level introduction will significantly reduce the rate of unfavourable treatment outcomes and reduce the burden of disease for national TB programmes. Thus, released funds can be used to strengthen country capacity to fight TB.

## 5. Conclusions

Increasing age, living in certain parts of the Republic, disability, pensioner status, unemployment, being HIV-positive, having pulmonary TB, and receiving category II treatment were independent factors that increased the risk of unfavorable treatment outcomes of patients who were initiated on first-line TB treatment in the Republic of Karakalpakstan. Factors associated with death included: being adult and elderly, living in certain parts of the Republic, disability, pensioner status, being HIV-positive, and receiving category II treatment. Factors associated with treatment failure included: being adolescent, female, and having pulmonary TB. Factors associated with loss to follow-up included: being male, disability, pensioner status, unemployment, and receiving category II treatment. In summary, there are sub-groups of patients who need special attention in order to decrease unfavourable treatment outcomes.

Our study identified these sub-groups of patients which need special attention during treatment, and this might help to increase favourable treatment outcomes. By using the study results, the Republican TB and Pulmonology Center based in Nukus can develop targeted interventions to address the risk factors causing unfavourable treatment outcomes. Particularly, the Republican TB and Pulmonology Center based in Nukus should endorse focused interventions based on the social characteristics of the patients. This study confirmed the results of a previous study [6] which reported high rates of failure among adolescents. Reasons for this are unclear and require further research, as well as interventions directed at this young group of patients. The study also has shown that two parallel health care systems like the TB and HIV programmes should strengthen their collaboration for better provision of TB and HIV care to patients.

## Figures and Tables

**Table 1:** Socio-demographic and clinical characteristics of tuberculosis patients between 2005 and 2020 in the Republic of Karakalpakstan, Uzbekistan.

Variable	*n* (%)
Total	35,122
Age in years	
Children (0–14)	2339 (7)
Adolescent (15–18)	2038 (6)
Adults (19–55)	24,394 (69)
Elderly (above 55)	6351 (18)
Sex	
Male	18,032 (51)
Female	17,090 (49)
Place of residence	
Urban	9289 (26)
Rural	19,774 (56)
Missing data	6059 (17)
Geographic distribution	
Central part	18,572 (53)
North-West	9060 (26)
South-East	7490 (21)
Social characteristics	
Worker	1605 (5)
Employee	1440 (4)
Pupil	2572 (7)
Disabled	819 (2)
Preschool age, kindergarten	125 (<1)
Preschool not attending kindergarten	671 (2)
Pensioner	3577 (10)
Unemployed	15,409 (44)
Missing data	8904 (25)
HIV status	
HIV positive	19 (<1)
HIV negative	26,373 (75)
Missing data	8730 (24)
Past prisoner	
No	2582 (7)
Yes	9 (<1)
Missing data	32,531 (92)
Contact with TB patient	
Yes	23,417 (67)
No	1915 (5)
Missing data	9790 (28)
TB type	
PTB (Pulmonary TB)	29,130 (83)
EPTB (Extrapulmonary TB)	5992 (17)
TB treatment category	
I category (2(3)HRZE(S)/4 H3R3) ^a^	27,465 (79)
II category (2HRZES/1(2)HRZE/5 H3R3E3) ^b^	7497 (21)
III category (2HRZ/4 H3R3) ^c^	160 (<1)

^a^ I treatment category—(a) new smear-positive pulmonary TB patients; (b) smear-negative pulmonary TB patients with large parenchymal lesions; (c) extrapulmonary TB patients with large lesions; (d) severe course of disease. Two-month, in some circumstances three-month, intensive phase with doses given daily followed by four-month continuation phase of treatment with doses given three times weekly. ^b^ II treatment category—(a) relapsed TB patients; (b) patients with treatment failure; (c) patients resuming treatment after loss to follow-up. Two-month, in some circumstances up to four-month, intensive phase with doses given daily followed by five-month continuation phase of treatment with doses given three times weekly. ^c^ III treatment category—(a) new smear-negative pulmonary TB patients with limited exposure; (b) extrapulmonary TB with limited exposure. Two-month intensive phase with doses given daily followed by four-month continuation phase of treatment with doses given three times weekly. H = isoniazid; R = rifampicin; Z = pyrazinamide; E = ethambutol; S = streptomycin.

**Table 2:** Treatment outcomes of tuberculosis patients, Republic of Karakalpakstan, 2005–2020 years.

Treatment Outcomes	*n* (%)
Favourable outcomes	27,682 (79)
Cured	6193 (18)
Treatment completed	21,489 (61)
Unfavourable outcomes	7440 (21)
Died	1419 (4)
Failure	1220 (3)
Loss to follow up	1948 (6)
Transfer out	2853 (8)

Cured = a patient who completed treatment with negative sputum smears; Treatment completed = a patient who completed treatment with no sputum smear examination result available at the end of treatment; Died = a patient who died before starting treatment or during the course of treatment; Failure = a patient whose treatment regimen was terminated or permanently changed to a new regimen or treatment strategy; Loss to follow up = a patient who did not start treatment or whose treatment was interrupted for two consecutive months or more; Transfer out = transferred to another treatment unit and no treatment outcome was assigned.

**Table 3:** Unfavourable outcomes among tuberculosis patients according to different socio-demographic and clinical characteristics, Republic of Karakalpakstan, Uzbekistan, 2005–2020.

Variable	*n*	Unfavourable Treatment Outcome		Univariable			Multivariable		
		*n*	%	RR	95%CI	*p* Value	aRR	95%CI	*p* Value
Age									
Children (0–14)	2339	186	8.0	0.36	(0.32–0.42)	<0.001	0.55	(0.43–0.69)	<0.001
Adolescent (15–18)	2038	394	19.3	0.88	(0.81–0.97)	<0.009	0.99	(0.85–1.1)	<0.838
Adults (19–55)	24,394	5331	21.9	1			1		
Elderly (above 55)	6351	1529	24.1	1.1	(1–1.16)	<0.001	1.27	(1.12–1.44)	<0.001
Sex									
Male	18,032	3816	21.2	1			1		
Female	17,090	3624	21.2	1	(0.96–1.04)	0.921	1.00	(0.95–1.05)	0.882
Place of residence									
Urban	9289	2191	23.6	1			1		
Rural	19,774	3466	17.5	0.74	(0.71–0.78)	<0.001	0.88	(0.84–0.93)	<0.001
Geographic variations									
South-East	7490	1077	14.4	1			1		
North-West	9060	1722	19.0	1.32	(1.23–1.42)	<0.001	1.29	(1.17–1.41)	<0.001
Central part	18,572	4641	25.0	1.74	(1.64–1.85)	<0.001	1.68	(1.55–1.83)	<0.001
Social characteristics									
Worker	1605	217	13.5	1			1		
Employee	1440	203	14.1	1.04	(0.87–1.25)	0.645	1.00	(0.84–1.20)	0.965
Pupil	2572	297	11.5	0.85	(0.73–1.03)	0.059	1.13	(0.92–1.40)	0.245
Disabled	819	185	22.6	1.67	(1.40–2.00)	0.000	1.49	(1.25–1.78)	<0.001
Preschool age, kindergarten	125	9	7.2	0.53	(0.28–1.01)	0.054	0.84	(0.44–1.61)	0.598
Preschool not attending kindergarten	671	44	6.6	0.49	(0.36–0.66)	0.000	0.90	(0.61–1.32)	0.581
Pensioner	3577	779	21.8	1.61	(1.40–1.85)	0.000	1.28	(1.07–1.53)	0.006
Unemployed	15,409	2981	19.3	1.43	(1.26–1.62)	0.000	1.38	(1.22–1.57)	<0.001
HIV status									
HIV positive	19	10	52.6	2.90	(1.89–4.45)	<0.001	3.16	(1.96–5.09)	<0.001
HIV negative	26,373	4782	18.1	1.00			1		
Contact with TB patient									
No	23,417	4068	17.4	1			1		
Yes	1915	391	20.4	1.18	(1.07–1.29)	0.001	1.20	(1.09–1.31)	0.011
TB type									
PTB(Pulmonary TB)	29,130	6712	23.0	1			1		
EPTB (Extrapulmonary TB)	5992	728	12.1	0.53	(0.49–0.57)	<0.001	0.67	(0.62–0.72)	<0.001
TB treatment category									
I category	27,465	5018	18.3	1			1		
II category	7497	2417	32.2	1.76	(1.69–1.84)	<0.001	1.22	(1.05–1.43)	0.011
III category	160	5	3.1	0.17	(0.07–0.41)	<0.001	0.26	(0.11–0.62)	0.002

TB, Tuberculosis; PTB, Pulmonary TB; EPTB, Extrapulmonary TB; RR, Risk Ratio.

**Table 4:** Deaths among tuberculosis patients according to different socio-demographic and clinical characteristics, Republic of Karakalpakstan, Uzbekistan, 2005–2020.

Variable	*n*	Deaths		Univariable			Multivariable		
		*n*	%	RR	95%CI	*p* Value	aRR	95%CI	*p* Value
Age									
Children (0–14)	2339	26	1.1	0.33	(0.22–0.48)	<0.001	0.48	(0.23–1.02)	0.056
Adolescent (15–18)	2038	40	2.0	0.58	(0.42–0.79)	0.001	0.69	(0.38–1.25)	0.225
Adults (19–55)	24,394	826	3.4	1			1		
Elderly (above 56)	6351	527	8.3	2.45	(2.20–2.72)	<0.001	2.35	(1.82–3.02)	<0.001
Sex									
Male	18,032	692	3.8	1			1		
Female	17,090	727	4.3	1.11	(1.00–1.23)	0.048	1.05	(0.91–1.21)	0.522
Place of residence									
Urban	9289	362	3.9	1					
Rural	19,774	727	3.7	0.94	(0.83–1.07)	0.356			
Geographic variations									
South-East	7490	281	3.8	1					
North-West	9060	348	3.8	1.02	(0.88–1.19)	0.765			
Central	18,572	790	4.3	1.13	(0.99–1.30)	0.065			
Social characteristics									
Worker	1605	32	2.0	1			1		
Employee	1440	21	1.5	0.73	(0.42–1.26)	0.261	0.66	(0.38–1.17)	0.161
Pupil	2572	22	0.9	0.43	(0.25–0.74)	0.002	0.80	(0.38–1.69)	0.565
Disabled	819	61	7.4	3.74	(2.46–5.68)	<0.001	2.93	(1.91–4.48)	<0.001
Preschool age, kindergarten	125	1	0.8	0.40	(0.056–2.91)	0.367	0.86	(0.10–7.13)	0.887
Preschool not attending kindergarten	671	6	0.9	0.45	(0.19–1.07)	0.070	1.08	(0.37–3.09)	0.892
Pensioner	3577	272	7.6	3.81	(2.66–5.48)	<0.001	1.76	(1.15–2.69)	0.009
Unemployed	15,409	381	2.5	1.24	(0.87–1.78)	0.237	1.11	(0.77–1.58)	0.578
HIV status									
HIV positive	19	5	26.3	8.40	(3.95–17.88)	<0.001	9.95	(4.76–20.80)	<0.001
HIV negative	26,373	826	3.1	1			1		
Contact with TB patient									
No	23,417	665	2.8	1					
Yes	1915	50	2.6	0.92	(0.69–1.22)	0.561			
TB type									
PTB(Pulmonary TB)	29,130	1296	4.4	1			1		
EPTB (Extrapulmonary TB)	5992	123	2.1	0.46	(0.38–0.55)	<0.001	0.72	(0.58–0.90)	<0.001
TB treatment category									
I category	27,465	800	2.9	1			1		
II category	7497	616	8.2	2.82	(2.55–3.12)	<0.001	3.27	(2.63–4.07)	<0.001
III category	160	3	1.9	0.64	(0.21–1.98)	0.442	1.27	(0.43–3.79)	0.669

TB, Tuberculosis; PTB, Pulmonary TB; EPTB, Extrapulmonary TB; RR, Risk Ratio.

**Table 5:** Treatment failure among tuberculosis patients according to different socio-demographic and clinical characteristics, Republic of Karakalpakstan, Uzbekistan 2005–2020.

Variable	*n*	Failure		Univariable			Multivariable		
		*n*	%	RR	95%CI	*p* Value	aRR	95%CI	*p* Value
Age									
Children (0–14)	2339	29	1.2	0.32	(0.22–0.46)	<0.001	0.76	(0.43–1.34)	0.343
Adolescent (15–18)	2038	115	5.6	1.44	(1.20–1.75)	<0.001	1.55	(1.12–2.15)	0.008
Adults (19–55)	24,394	952	3.9	1			1		
Elderly (above 56)	6351	124	2.0	0.50	(0.42–0.60)	0.027	0.67	(0.44–1.03)	0.069
Sex									
Male	18,032	541	3.0	1			1		
Female	17,090	679	4.0	1.32	(1.19–4.48)	<0.001	1.29	(1.11–1.49)	0.001
Place of residence									
Urban	9289	331	3.6	1					
Rural	19,774	711	3.6	1.01	(0.89–1.15)	0.890			
Geographic variations									
South-East	7490	234	3.1	1			1		
North-West	9060	380	4.2	1.34	(1.14–1.58)	<0.001	1.21	(0.98–1.49)	0.069
Central	18,572	606	3.3	1.04	(0.90–1.21)	0.566	0.93	(0.77–1.12)	0.443
Social characteristics									
Worker	1605	45	2.8	1			1		
Employee	1440	48	3.3	1.19	(0.79–1.77)	0.397	1.07	(0.72–1.60)	0.729
Pupil	2572	70	2.7	0.97	(0.67–1.4)	0.875	0.87	(0.53–1.41)	0.571
Disabled	819	25	3.1	1.09	(0.67–1.76)	0.729	1.05	(0.65–1.71)	0.834
Preschool age, kindergarten	125	1	0.8	0.29	(0.03–2.05)	0.213	0.43	(0.06–3.21)	0.409
Preschool not attending kindergarten	671	1	0.1	0.05	(0–0.38)	0.004	0.10	(0.01–0.78)	0.028
Pensioner	3577	78	2.2	0.78	(0.54–1.11)	0.174	0.97	(0.58–1.62)	0.898
Unemployed	15,409	519	3.4	1.20	(0.88–1.62)	0.231	1.05	(0.77–1.42)	0.772
HIV status									
HIV positive	19	0	0.0	0.00	(0.00–0.00)	<0.001	0.00	(0.00–0.00)	<0.001
HIV negative	26,373	789	3.0	1					
Contact with TB patient									
No	23,417	630	2.7	1			1		
Yes	1915	105	5.5	2.04	(1.67–2.49)	<0.001	1.94	(1.58–2.39)	<0.001
TB type									
PTB (Pulmonary TB)	29,130	1200	4.1	1			1		
EPTB (Extrapulmonary TB)	5992	20	0.3	0.08	(0.05–0.13)	<0.001	0.10	(0.06–0.17)	<0.001
TB treatment category									
I category	27,465	730	2.7	1			1		
II category	7497	490	6.5	2.46			1.34	(0.87–2.07)	0.179
III category	160	0	0.0	0.00	(0.00–0.00)	<0.001	0.00	(0.00–0.00)	<0.001

TB, Tuberculosis; PTB, Pulmonary TB; EPTB, Extrapulmonary TB; RR, Risk Ratio.

**Table 6:** Loss to follow up among tuberculosis patients according to different socio-demographic and clinical characteristics, Republic of Karakalpakstan, Uzbekistan 2005–2020.

Variable	*n*	Loss to Follow Up		Univariable			Multivariable		
		*n*	%	RR	95%CI	*p* Value	aRR	95%CI	*p* Value
Age									
Children (0–14)	2339	84	3.6	0.63	(0.51–0.78)	<0.001	0.97	(0.64–1.47)	0.879
Adolescent (15–18)	2038	74	3.6	0.63	(0.50–0.80)	0.944	0.86	(0.63–1.17)	0.331
Adults (19–55)	24,394	1397	5.7	1			1		
Elderly (above 56)	6351	393	6.2	1.08	(0.97–1.20)	<0.001	1.16	(0.91–1.47)	0.237
Sex									
Male	18,032	1092	6.1	1			1		
Female	17,090	856	5.0	0.83	(0.75–0.90)	<0.001	0.81	(0.72–0.90)	<0.001
Place of residence									
Urban	9289	568	6.1	1			1		
Rural	19,774	881	4.5	0.73	(0.66–0.81)	<0.001	0.88	(0.78–0.99)	0.036
Geographic variations									
South-East	7490	279	3.7	1			1		
North-West	9060	430	4.7	1.01	(0.8–1.27)	0.924	1.17	(0.96–1.43)	0.112
Central	18,572	1239	6.7	0.89	(0.76–1.02)	0.116	1.78	(1.50–2.11)	<0.001
Social characteristics									
Worker	1605	42	2.6	1			1		
Employee	1440	32	2.2	0.85	(0.53–1.33)	0.481	0.87	(0.55–1.36)	0.535
Pupil	2572	71	2.8	1.05	(0.72–1.53)	0.781	1.16	(0.73–1.86)	0.527
Disabled	819	43	5.3	2.01	(1.32–3.04)	0.001	1.82	(0.20–2.76)	0.005
Preschool age, kindergarten	125	3	2.4	0.92	(0.28–2.91)	0.884	0.89	(0.27–2.91)	0.847
Preschool not attending kindergarten	671	32	4.8	1.82	(1.16–2.86)	0.009	1.76	(0.96–3.22)	0.067
Pensioner	3577	168	4.7	1.79	(1.28–2.50)	0.001	1.67	(1.13–2.46)	0.010
Unemployed	15,409	786	5.1	1.95	(1.43–2.64)	<0.001	1.95	(1.43–2.65)	<0.001
HIV status									
HIV positive	19	2	10.5	1.40	(0.64–8.90)	0.192			
HIV negative	26,373	1159	4.4	1					
Contact with TB patient									
No	23,417	1031	4.4	1					
Yes	1915	73	3.8	0.87	(0.69–1.09)	0.225			
TB type									
PTB (Pulmonary TB)	29,130	1623	5.6	1					
EPTB (Extrapulmonary TB)	5992	325	5.4	0.97	(0.87–1.09)	0.649			
TB treatment category									
I category	27,465	1282	4.7	1			1		
II category	7497	664	8.9	1.90	(1.73–2.08)	<0.001	1.60	(1.32–1.95)	<0.001
III category	160	2	1.3	0.27	(0.07–1.06)	0.061	0.34	(0.08–1.34)	0.122

TB, Tuberculosis; PTB, Pulmonary TB; EPTB, Extrapulmonary TB; RR, Rate Ratio.

## Data Availability

The data that support the findings of this study are available from the corresponding author, (J.G.), upon reasonable request.
